# Determination of Biotransformation Products of Platinum Drugs in Rat and Human Urine

**DOI:** 10.1155/MBD.1997.97

**Published:** 1997

**Authors:** Xia Tang, Jerry W. Hayes, II, Louis Schroder, William Cacini, John Dorsey, R. C. Elder, Katherine Tepperman

**Affiliations:** 1 Barrett Center for Cancer Prevention, Treatment and Research Cincinnati OH 45267 USA; 2 College of Pharmacy University of Cincinnati Cincinnati OH 45267 USA; 3 Department of Chemistry Florida State University Tallahassee FL 323006-30006 USA; 4 Biomedical Chemistry Research Center Department of Chemistry University of Cincinnati Cincinnati OH 45221-0172 USA

## Abstract

Cisplatin is an extremely effective cancer chemotherapeutic agent, but its use is often accompanied by toxicity. Second generation drugs such as carboplatin are becoming more widely used because of reduced toxicity. Since biotransformation products have been implicated in the toxic responses, we have begun to investigate the reactions of cisplatin and carboplatin with potential biological ligands. Reaction products were characterized using HPLC with inductively coupled plasma - mass spectrometry (HPLC-ICP-MS), ^1^H and ^13^C NMR and fast atom bombardment - mass spectrometry (FAB-MS). Three Pt-creatinine complexes, *cis*-[Pt(NH_3_)_2_Cl(Creat)]^+^, *cis*-[Pt(NH_3_)_2_(H_2_O)(Creat)]^2+^ and *cis*-[Pt(NH_3_)_2_(Creat)_2_]^2+^, were synthesized and the platinum was shown to coordinate to the ring nitrogen, N(3). Human urine samples from patients on cisplatin chemotherapy were shown to contain cisplatin, its hydrolysis product and biotransformation products containing Pt-creatinine, Pt-urea and Pt-uric acid complexes. Urine from carboplatin patients shows fewer biotransformation products. Studies with control and diabetic (protected against cisplatin toxicity) rats showed systematic differences in the biotransformation products formed on administration of cisplatin.


### DETERMINATION OF BIOTRANSFORMATION PRODUCTS OF PLATINUM DRUGS IN RAT AND HUMAN URINE

Xia Tang[1], Jerry W. Hayes, II[1], Louis Schroder[1], William Cacini[2]

John Dorsey[3], R. C. Elder[4], and Katherine Tepperman*[4]

[1] Barrett Center for Cancer Prevention, Treatment and Research, Cincinnati, OH 45267, USA
[2] College of Pharmacy, University of Cincinnati, Cincinnati, OH 45267, USA
[3] Department of Chemistry, Florida State University, Tallahassee, FL 323006-30006, USA
[4] Biomedical Chemistry Research Center, Department of Chemistry, University of Cincinnati, Cincinnati, OH 45221-0172, USA

### ABSTRACT

Cisplatin is an extremely effective cancer chemotherapeutic agent, but its use is often accompanied by toxicity. Second generation drugs such as carboplatin are becoming more widely used because of reduced toxicity. Since biotransformation products have been implicated in the toxic responses, we have begun to investigate the reactions of cisplatin and carboplatin with potential biological ligands. Reaction products were characterized using HPLC with inductively coupled plasma - mass spectrometry (HPLC-ICP-MS), $^{1}H$ and $^{13}C$ NMR and fast atom bombardment - mass spectrometry (FAB-MS). Three Pt-creatinine complexes, *cis*-$[Pt(NH_3)_2Cl(Creat)]^{+}$, *cis*-$[Pt(NH_3)_2(H_2O)(Creat)]^{2+}$ and *cis*-$Pt(NH_3)_2(Creat)_2]^{2+}$, were synthesized and the platinum was shown to coordinate to the ring nitrogen, N(3). Human urine samples from patients on cisplatin chemotherapy were shown to contain cisplatin, its hydrolysis product and biotransformation products containing Pt-creatinine, Pt-urea and Pt-uric acid complexes. Urine from carboplatin patients shows fewer biotransformation products. Studies with control and diabetic (protected against cisplatin toxicity) rats showed systematic differences in the biotransformation products formed on administration of cisplatin.

### INTRODUCTION

Cisplatin, *cis*-diamminedichloroplatinum(II), is one of the most widely used and most successful cancer chemotherapeutic agents (1,2). Cisplatin is most effective in the treatment of metastatic testicular tumors, but has been used either alone or in combination protocols for the treatment of a number of other types of tumors (3). There is strong evidence to suggest that DNA represents the principal target for cisplatin in the cell (2), however, the interaction of cisplatin with other biological molecules has been less well studied. The clinical application of cisplatin is accompanied by significant toxicity, of which nephrotoxicity is dose limiting (4). Although the mechanism of nephrotoxicity is still unknown, the biotransformation products of cisplatin are believed to play a role (5). A number of "second generation" platinum complexes, such as carboplatin, have been developed in an attempt to overcome the toxicity problems experienced by cisplatin patients (see reference 6 for a review). The observed reduction of toxicity with carboplatin, which may be a function of its lower rate and degree of biotransformation, has resulted in its increasing use in clinical treatment protocols (7).

Previous studies have identified Pt-methionine complexes in rat plasma after cisplatin treatment (8). Also, there is evidence suggesting the formation of Pt complexes with cysteine and glutathione in kidney cytosol and urine of rats treated with cisplatin (9). The reactions of cisplatin with S-containing compounds such as methionine, cysteine and glutathione, have been extensively investigated using HPLC, mass spectrometry (MS) and NMR (10-13). However, few studies with other biological ligands such as creatinine have been reported. Creatinine is an end product of creatine metabolism, whose concentration in serum and urine is used as an indicator of renal function. Elevated blood levels of creatinine are indicative of kidney damage (14). Geraldes *et al.* found that several dimeric Pt-creatinine complexes formed in the 1:1 reaction of *cis*-diammine-diaquaplatinum(II) with creatinine (15). Various products were also obtained when $PtX_4^{2-}$ (where X is Cl (16, 17) or $NO_2$ (17, 18)) reacted with creatinine.

Previously we have used HPLC interfaced with inductively coupled plasma - mass spectrometry (HPLC-ICP-MS) to investigate various biotransformation products of gold antiarthritis drugs (19) and possible products of cisplatin reactions with sulfur ligands such as cysteine and methionine (20). Here we describe the reactions of cisplatin with nitrogenous ligands such as creatinine, urea and uric acid. Further characterization of the creatinine containing products has been obtained using fast atom bombardment - mass spectrometry, $^1H$ and $^{13}C$ NMR spectrometry. Urine specimens from human patients undergoing cisplatin or carboplatin chemotherapy have been examined and some biotransformation products identified. Rats have been treated with streptozotocin (STZ) to make them diabetic and apparently create some level of protection from the nephrotoxicity associated with administration of high doses of cisplatin (21). Rat urine specimens have been examined and appear to show some systematic differences in biotransformation products. We report those details here.

### MATERIALS AND METHODS

**Chemicals.** Cisplatin was purchased from Johnson Matthey (Ward Hill, MA). Creatinine (Sigma), urea (Fisher Scientific, >99%) and uric acid (Aldrich, >99%) were used upon receipt without further purification. All other chemicals were obtained from various commercial sources and used as received. Water used in the preparation of mobile phase and other solutions was purified (18 MΩ) with a Barnstead Nanopure system (Milford, MA) equipped with a 0.2 μm filter.

**HPLC.** Two HPLC systems were used during the course of this work, a Waters (Milford, MA) model 600 E Multisolvent Delivery System or a model 510 isocratic system. Samples were injected using Rheodyne (Cotati, CA) 7125 injectors equipped with 20 or 50 μL sample loops. Separations were performed on a Rainin (Woburn, MA) Microsorb-MV $C_{18}$ column with 250 mm x 4.6 mm I.D. and 5 μm particle size. A Hamilton (Reno, Nevada) PRP-1 column with 150 mm x 4.1 mm I.D. and 5 μm particle size was used to confirm the identity of several sample peaks with known materials. An Alltech (Deerfield, IL) Adsorbsphere $C_{18}$ cartridge guard column (10 mm x4.6 mm I.D., 5 μm particle size) or a Rainin Dynamax guard column (45 mm x 4.6 mm I.D., 5 μm particle size) were also used. The two mobile phase systems used throughout the experiments were similar to that described by Zhao *et al.*(20). One consisted of 0.1% trifluoroacetic acid (TFA), 1.0 mM 1-heptanesulfonate and 15% methanol. A second mobile phase used 0.1% TFA, 7.0 mM 1-heptanesulfonate and 5% methanol. The pH for each was adjusted to 2.6-2.7 using NaOH solution. Prior to use, the mobile phase was filtered through a 0.45 μm Nylon-66 membrane and ultrasonicated to eliminate dissolved gasses. All separations were performed in an isocratic mode with a flow rate of 1.0 ml/min. Chromatographic conditions were altered for some experiments to facilitate the observation of species other than the hydrolysis products (*i.e.* products of the reactions with creatinine or urea). Retention characteristics of aquated products were vetted for each set of chromatographic conditions including the addition of $AgNO_3$ to change the relative amounts of the hydrolysis products.

**ICP-MS.** Eluent from the HPLC column was analyzed on-line with a Sciex Elan 250 inductively coupled plasma - mass spectrometer (Toronto, Ontario). The column was coupled to the instrument via PTFE capillary tubing (96 cm x 0.1 mm I.D.). The tubing has a volume of approximately 0.08 μL/cm and has a minimal effect on extra-column peak broadening (22). The instrument was operated in selected ion detection mode and platinum, m/z 195, was monitored.

Typical operating parameters for the ICP-MS were: RF power, 1.25-1.4 kW; nebulizer argon flow rate, 0.4 L/min; auxiliary argon flow rate, 1.1 L/min; coolant flow rate, 14 L/min; spray chamber, 2-5°C.

**NMR Spectra.** The $^1H$ (250.15 MHz) and $^{13}C$ (62.90 MHz) NMR spectra were recorded on a Brüker AC 250 spectrometer. $^1H$ spectra were referenced to 2,2-dimethylsilapentanesulfonate (DSS) as an internal standard. The free creatinine sample was prepared by dissolving creatinine (0.0086 mmol) in 1 mL of $H_2O$. The 1:1 reaction mixture of cisplatin and creatinine was prepared by incubating 0.0086 mmol cisplatin with 0.0086 mmol creatinine in 1 mL of $H_2O$ for 3 h at 47°C. The 1:2 reaction mixture was prepared by incubating 0.0086 mmol cisplatin with 0.0174 mmol creatinine in 1 ml of $H_2O$ for 24 h at 47°C. Phosphate buffer was not used in these experiments due to the concern that cisplatin may react with phosphate to form phosphato complexes (23, 24). $D_2O$ (20%) was added to all samples to ensure a deuterium lock and continuous wave decoupling was employed to suppress the $H_2O$ resonance.

Composite pulse decoupling (WALTZ-16) $^{13}C$ spectra were recorded with respect to tetramethylammonium chloride (TMA) as an external standard. The chemical shift of TMA in $D_2O$ was measured relative to tetramethylsilane in $CD_3Cl$ using a concentric arrangement. Data acquisition was for 20% $D_2O$ solutions of free creatinine or the 1:2 Pt-creatinine complex.

**FAB-MS Spectra.** These spectra were obtained on a VG 30-250 mass spectrometer. 3-Nitrobenzyl alcohol was used as matrix and the spectra were detected using positive ion detection mode. The 1:1 reaction products of cisplatin and creatinine were formed by incubating 0.083 mmol cisplatin and 0.083 mmol creatinine in 10 mL of water for 3 h at 47°C. The reaction solution was concentrated by lyophilization and then redissolving in a small volume of water (several μL). The 1:2 reaction products were formed by incubating 0.083 mmol cisplatin with 0.166 mmol creatinine in 2.4 mL of aqueous solution for 24 h at 47°C. After incubation, 0.31 mmol $Li_2SO_4$ was added to the solution and then ethanol was introduced dropwise until a white precipitate formed. The precipitate was collected by vacuum filtration, washed with methanol and analyzed by mixing the solid with the matrix at the probe tip.

**Preparation of Reaction Solutions for HPLC Analysis.** The cisplatin hydrolysis products were prepared by incubating 0.001 mmol cisplatin in 5 mL of saline solution (0.9% NaCl) at 37°C for 12 hours. Diamminediaquaplatinum(II) was prepared by incubating 0.005 mmol cisplatin with a stoichiometric amount of silver nitrate in 5 mL water in absence of light for 12 h at room temperature. The solution was then filtered (0.45 μm, Polysulfone) to remove the AgCl precipitate.

The preparation of the 1:1 reaction mixture of cisplatin and creatinine is described above. The reaction products of cisplatin and urea were formed by incubating cisplatin (0.002 mmol) with urea (3.33 mmol) in 10 mL of water for 5 h at 37°C. The reaction of cisplatin and uric acid was studied by incubating cisplatin (0.002 mmol) with uric acid (0.018 mmol) in 10 mL of water for 6 h at 37°C. The solutions of platinum complexes were diluted to the desired concentration with mobile phase immediately prior to analysis.

**Urine Samples.** In some experiments, male Sprague-Dawley rats were injected with cisplatin (5 mg/kg, intravenous) and immediately placed in metabolism cages arranged for collection of feces-free urine. After collecting for 2 hours, the urine was immediately analyzed. Urine from STZ-diabetic as well as normal rats was collected (25). Human urine specimens were collected from patients receiving cisplatin or carboplatin chemotherapy (26). The clinical urine samples were either analyzed immediately after collection or divided into 1.5 mL aliquots, flash frozen with liquid nitrogen and stored in the absence of light at -20°C. Specimens were thawed rapidly using a method described by Kinoshita (27). All urine samples were filtered through 0.45 μm polysulfone filters and diluted with mobile phase prior to analysis.

### RESULTS

**Reactions of cisplatin with creatinine.** One goal of these experiments was to develop methods to identify biotransformation products of cisplatin, since it is believed that these products may contribute to the toxicity observed with the drug (5). In aqueous solution, the two labile chloride ligands in cisplatin can be displaced by water or hydroxyl ion. Figure 1a shows the HPLC-ICP-MS separation of cisplatin (C) and its hydrolysis products. Both the monoaqua ($H_1$) and diaqua ($H_2$) hydrolysis products are formed after incubation in saline solution for 12 h at 37°C. These

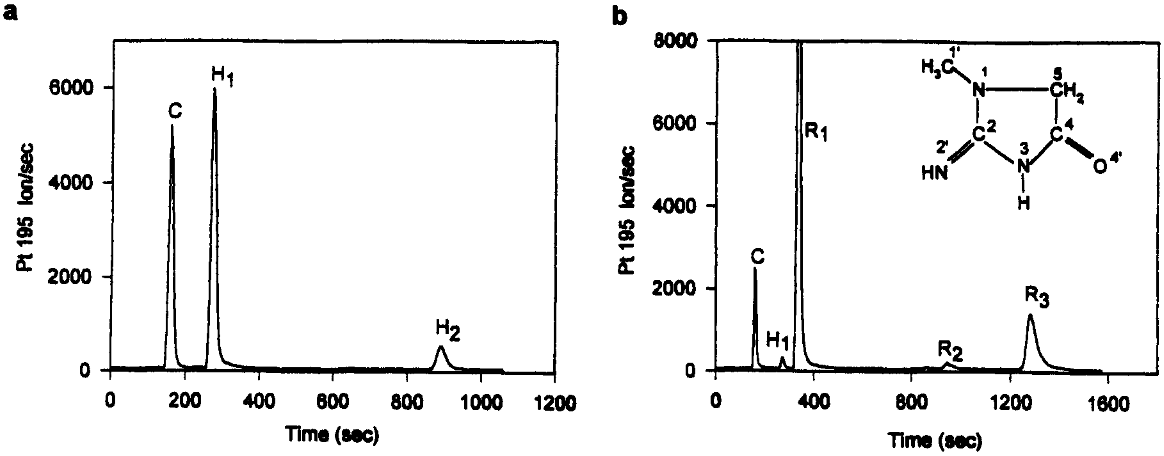

**Figure 1 (a) HPLC-ICP-MS separation of cisplatin (C), mono-aqua ($H_1$) and diaqua hydrolysis ($H_2$) products. Mobile phase conditions: 0.1% trifluoroacetic acid (TFA), 1 mM 1-heptanesulfonate, 15% methanol, pH 2.6-2.7. Column: Rainin Microsorb-MV $C_{18}$. The ICP-MS was set to monitor Pt at m/z 195. (b) HPLC separation of 1:1 reaction mixture of cisplatin and creatinine. Three Pt-creatinine complexes (peak $R_1$,$R_2$ and $R_3$) are formed. LC conditions as in (a). The insert shows the structural formula of creatinine.**

hydrolysis products were identified by adding $AgNO_3$ to cisplatin in aqueous solution to drive the ligand exchange equilibrium toward the formation of the diaqua species. A subsequent chromatogram of this solution thus permitted the identification of the diaqua hydrolysis product.

Cisplatin may undergo reactions with various biological nucleophiles resulting in biotransformation products which may have activity and toxicity different from that of the parent drug. Creatinine is an end product of creatine metabolism, whose concentration in serum and urine is used as an indicator of renal function. Elevated blood levels of creatinine are indicative of kidney damage (14). The presence of several electron donor groups on creatinine provides multiple ligation sites for platinum. HPLC separation of the 1:1 reaction mixture of cisplatin and creatinine is shown in Figure 1b. Cisplatin (C), the monoaqua hydrolysis product ($H_1$) and three Pt-creatinine complexes (peak $R_1$,$R_2$ and $R_3$) are observed. Peak $R_1$ is the dominant component. Under these reaction conditions little, if any of the diaqua hydrolysis product is produced.

To further characterize the three Pt-creatinine complexes, $^1H$ NMR was used to monitor the reaction mixtures obtained at different molar ratios of cisplatin and creatinine. Figure 2 shows the spectra of free creatinine and the 1:1 and 1:2 reaction solutions of cisplatin and creatinine. The chemical shifts of the $CH_3$ and $CH_2$ protons in free creatinine are 3.03 and 4.04 ppm respectively (Figure 2a). Two new resonances of the $CH_3$ protons are observed shifted down field by 0.05 and 0.08 ppm upon complexation with platinum, however only one new signal (III) from $CH_2$ is observed. This may be due to peak overlapping. In the 1:1 reaction solution (Figure 2b), signal I is the major resonance of $CH_3$ protons. In Figure 2c, signal II becomes the dominant resonance

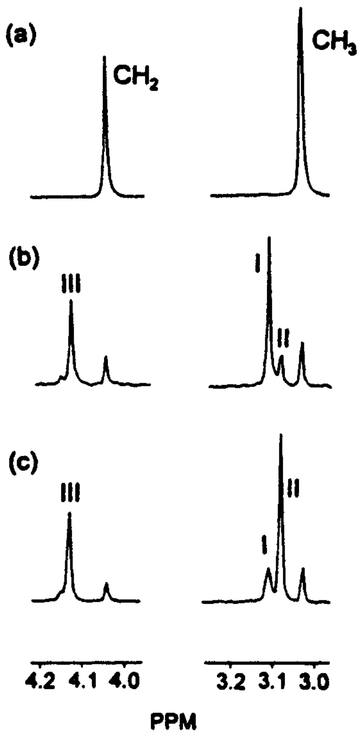

**Figure 2 $^1H$ NMR spectra of (a) free creatinine (b) 1:1 and (c) 1:2 reaction mixture of cisplatin and creatinine.**

when cisplatin reacts with creatinine at a 1:2 molar ratio. This change suggests that signal I results from a 1:1 platinum-creatinine complex and signal II from a 1:2 platinum-creatinine complex.

The mass spectrum of the 1:1 reaction mixture of cisplatin and creatinine is shown in Figure 3. The molecular ions of the 1:1 Pt-creatinine complex, *cis*-$[Pt(NH_3)_2Cl(Creat)]^+$, and the 1:2 Pt-creatinine complex, *cis*-$[Pt(NH_3)_2(Creat)_2+H]^+$ are observed (peaks at 376-380 and 453-457 respectively). Here, the 1:2 complex loses H, forming the $[M-H]^+$ ion. The peak intensities in m/z 376-380 and 453-457 regions are compared with the theoretical isotopic abundance calculated from molecular formulas (Table I). It can be seen that the intensity distributions in both regions match their corresponding theoretical abundance. In addition, the peaks at m/z 359-363 correspond to the loss of one $NH_3$ from the *cis*-$[Pt(NH_3)_2Cl(Creat)]^+$ ion and signals at m/z 436-438 and 419-421 result from the *cis*-$Pt(NH_3)_2(Creat)_2$-$H]^+$ ion losing one and two $NH_3$ respectively. These observations confirm that the peaks at m/z 376-380 are the molecular ions of the 1:1 Pt-creatinine complex with one platinum and one chloride present in its structure. The group of peaks at m/z 453-457 correspond to the 1:2 complex formed by displacing the two chloride ligands of cisplatin with creatinine.

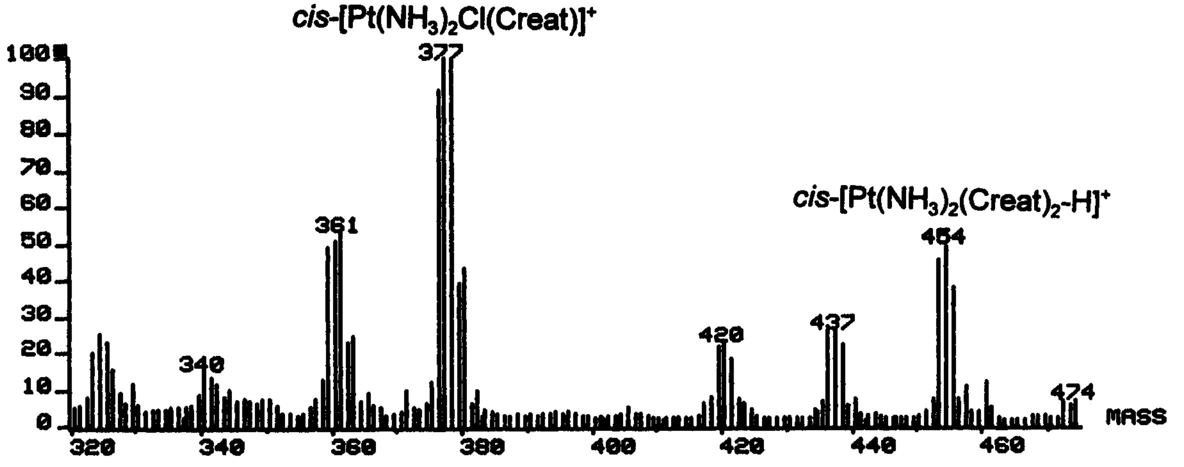

**Figure 3 FAB-mass spectrum of the 1:1 reaction mixture of cisplatin and creatinine.**

Using this mass spectrum, we conclude that peak 1 in Figure 1a is the 1:1 Pt-creatinine complex: *cis*-$[Pt(NH_3)_2Cl(Creat)]^+$. To identify the other two Pt-creatinine complexes, The 1:2 Pt-creatinine complex was synthesized and isolated from the reaction solution by sulfate precipitation. The chromatogram of this complex shows a dominant peak $R_3$, with a small amount of the 1:1 complex (peak $R_1$) present as an impurity. The mass spectrum of this solid product (Figure 4) shows a molecular ion cluster with the most intense peak at m/z 552, which corresponds to *cis*-$[Pt(NH_3)_2(Creat)_2SO_4{+}H]^+$. Thus peak $R_3$ in Figure 1a is identified as the 1:2 Pt-creatinine complex, *cis*-$[Pt(NH_3)_2(Creat)_2]^{2+}$. When this 1:2 complex was incubated in water for 6 h, peak $R_3$ remained unchanged, peak $R_1$ decreased and peak $R_2$ appeared at ca. 940s. Thus peak $R_2$ is likely to be the 1:1 complex, *cis*-$[Pt(NH_3)_2(H_2O)(Creat)]^{2+}$, formed from *cis*-$[Pt(NH_3)_2Cl(Creat)]^+$ via exchange of chloride with water.

**Table I Masses and relative intensities of *cis*- $[Pt(NH_3)_2Cl(Creat)]^+$ and *cis*-$[Pt(NH_3)_2(Creat)_2]^{2+}$**

(a) *cis*-$[Pt(NH_3)_2Cl(Creat)^+$ , m/z 376-380

| Mass (m/z) | Relative intensity (%) (Measured) | Relative abundance (%) (Calculated) |
|---|---|---|
| 376 | 91.3 | 86.8 |
| 377 | 100.0 | 94.3 |
| 378 | 99.9 | 100.0 |
| 379 | 39.5 | 34.9 |
| 380 | 43.3 | 42.3 |

(b) *cis* -$[Pt(NH_3)_2(Creat)_2 - H]^+$, m/z 453-457

| Mass (m/z) | Relative intensity (%) (Measured) | Relative abundance (%) (Calculated) |
|---|---|---|
| 453 | 92.7 | 86.9 |
| 454 | 100.0 | 100.0 |
| 455 | 77.9 | 78.8 |
| 456 | | 9.4 |
| 457 | 21.6 | 19.8 |

* The signal of m/z 456 is not differentiated from base line.

To determine the Pt-coordination site on creatinine, $^{13}C$ NMR spectra of free creatinine and the 1:2 Pt-creatinine complex were measured. The chemical shifts of the $C_{1'}$, $C_2$, $C_4$ and $C_5$ of creatinine are 30.36, 169.51, 189.09 and 56.60 ppm respectively. The assignments are based on previous work (15, 28). The resonances of the 1:2 Pt-creatinine complex occur at 31.42, 164.36, 182.55 and 55.00 ppm respectively (29). The chemical shifts of $C_2$ and $C_4$ show large changes (5.15 and 6.54 ppm respectively), while signals from $C_{1'}$ and $C_5$ shift slightly (1.06 and 1.60 ppm respectively). These data suggest that creatinine forms complexes with platinum through the cyclic nitrogen N(3). This is consistent with the structure of *cis* -$[Pt(Creat)_2I_2]{\bullet}3H_2O$ determined by X-ray crystallography (30).

**Reactions of urea and uric acid with cisplatin.** Based on their structures and relative abundances in urine, urea and uric acid were incubated with cisplatin to determine whether new platinum containing products formed. The reaction of cisplatin and urea produced three platinum complexes (Figure 5a). The major product (ca. 675 s) is adjacent to the monoaqua hydrolysis product and is not completely separated from it. In addition two minor products are observed at ca. 810 and 910 s. An additional byproduct of cisplatin hydrolysis is also observed at ca. 240 s and has not yet been identified. In this separation, a higher concentration of the ion pairing agent and a lower methanol concentration were employed to improve the resolution of overlapping peaks. The diaqua hydrolysis product is retained by the column and is not observed under this chromatographic condition.

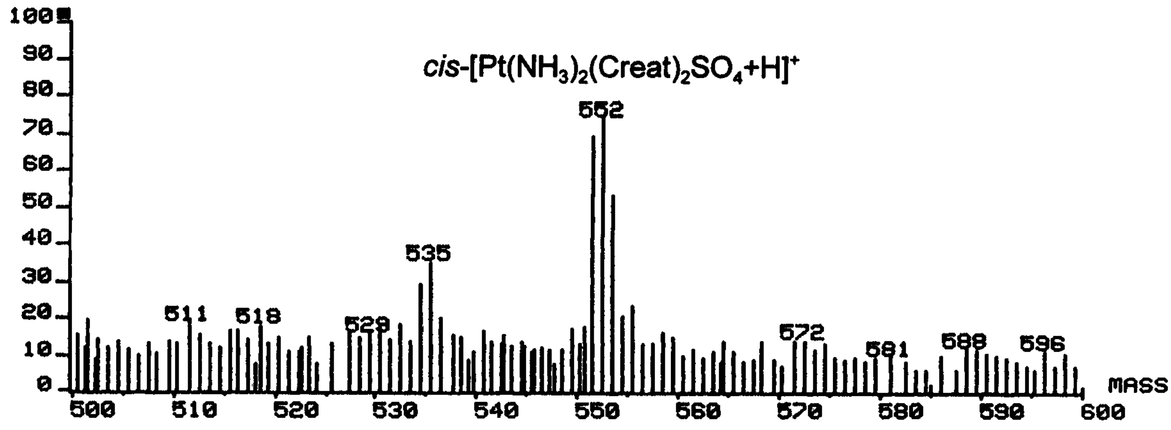

**Figure 4** FAB-mass spectrum of the 1:2 complex of cisplatin and creatinine, *cis*-$[Pt(NH_3)_2(Creat)_2]SO_4$.

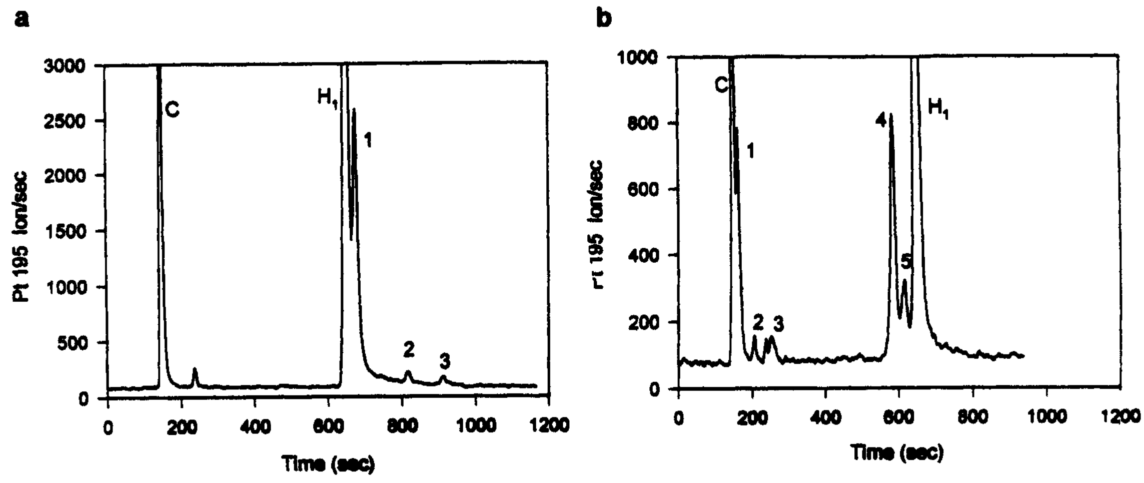

**Figure 5** HPLC separation of the reaction mixture of cisplatin and (a) urea and (b) Uric acid. The reaction of cisplatin and urea produced three Pt-urea complexes: the major product (peak 1: ca. 675 s) and two minor components at ca. 810 (peak 2) and 910 s (peak 3). Incubation of cisplatin and uric acid also showed the formation of several new species (peaks 1-5). Mobile phase condition: 0.1% TFA, 7 mM 1-heptanesulfonate, 5% methanol, pH 2.6-2.7. Column: Rainin Microsorb-MV $C_{18}$. The retention times for cisplatin (C) and the monoaqua hydrolysis product ($H_1$) are indicated.

The incubation of cisplatin and uric acid also resulted in the formation of several new platinum species (Figure 5b). Again, although complete separation of all of the components is not attained, qualitative identifications can be made. By changing the mobile phase conditions slightly (10% methanol) and using on-line UV detection, peak 4 has been determined to contain an additional minor component not shown here. Further characterization of these products is in progress.

**Identification of Biotransformation Products in Rat Urine.** Rats treated with streptozotozin (STZ) suffer pancreatic damage and develop a diabetic condition (31). Interestingly, these animals, when compared to control rats, are resistant to the kidney damage which accompanies high doses of cisplatin (32). The mechanism of the protection is not known. We examined urine

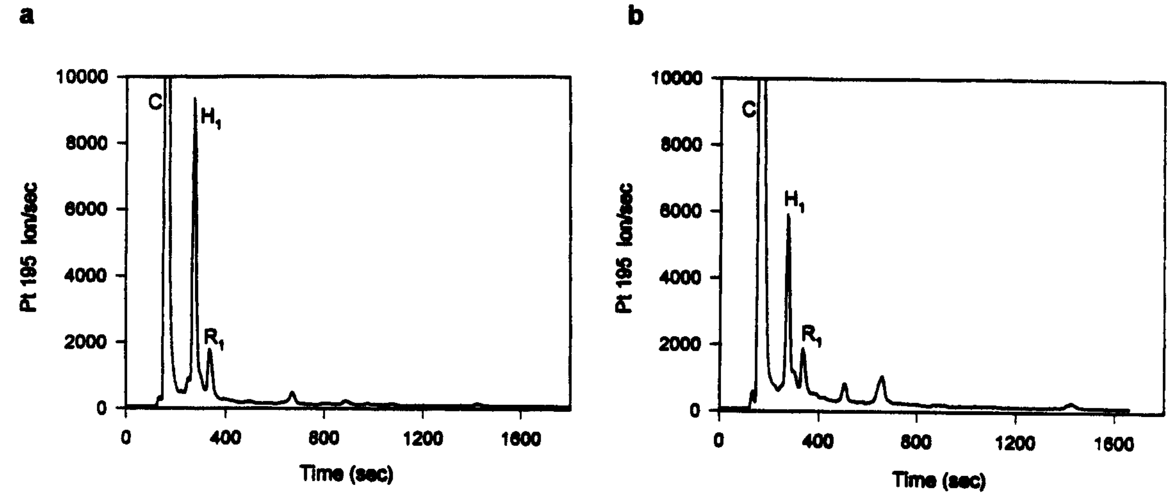

**Figure 6 HPLC separation of urine from (a) control and (b) diabetic rat treated with cisplatin. The major component is cisplatin. Several platinum species are observed. The retention times of two species agree with that of the monoaqua hydrolysis product and the 1:1 Pt-creatinine complex, *cis*-$[Pt(NH_3)_2Cl(Creat)]^+$. LC conditions as in Figure 1. Retention times for cisplatin (C), the monoaqua hydrolysis product ($H_1$) and the 1:1 reaction product of cisplatin and creatinine ($R_1$) are indicated.**

specimens from both diabetic and control rats injected with cisplatin (5 mg/kg). The HPLC-ICP-MS chromatograms are shown in Figure 6. The major component is cisplatin, but several other platinum containing species are observed as well. Two of the species show retention times expected for the monoaqua hydrolysis product and the 1:1 Pt-creatinine complex, *cis*-$[Pt(NH_3)_2Cl(Creat)]^+$.

The identity of the Pt-creatinine complex was confirmed in two ways. First, a similar quantity of the synthetic complex was added to the rat urine specimen. The peak attributed to the complex grew in size but did not change shape, indicating an exact equivalence in retention times between the known complex and that in the rat urine specimen. Second, further evidence for the identity of the 1:1 Pt-creatinine complex in rat urine was provided by separating both the urine sample and the 1:1 reaction mixture of cisplatin and creatinine on a PRP-1 column. The stationary phase of the PRP-1 column is a polystyrene-divinylbezene copolymer. The retention mechanism of the PRP-1 column is based on adsorption, which differs from the partition mechanism of a regular $C_{18}$ column. Therefore any peak coelution may be tested by chromatographing each of the samples on two different columns. Again, one of the platinum containing species in the rat urine elutes at the same retention time as this 1:1 complex, which implies that *cis* -$[Pt(NH_3)_2Cl(Creat)]^+$ is present.

Figure 6a shows the chromatogram of urine from a control rat, whereas that in Figure 6b is from a diabetic rat. In both cases the overall distribution of peaks is quite similar; however, there is an additional peak in Figure 6b (ca. 500 s) which was also present in a urine specimen from a second diabetic rat and missing from the urine specimens of four different control rats treated with cisplatin. The identity of this peak is under study. We currently do not know whether this is a "protective" species. It is clearly a minor component of the total platinum present in urine.

**Cisplatin and Its Biotransformation Products in Human Urine.** Having identified specific biotransformation products in the urine of rats, we monitored urine samples from a number of cancer patients to determine the platinum species present. Figure 7 shows the platinum species in urine taken from a patient being treated with cisplatin for lung cancer. The peak for cisplatin corresponds to the major component. There is a significant contribution from the monoaqua hydrolysis product of the drug as well. More interestingly the second most abundant platinum-containing species is the 1:1 Pt-creatinine complex: *cis*-$[Pt(NH_3)_2Cl(Creat)]^+$. A peak which is not completely separated from the monoaqua hydrolysis product at ca. 675 s has the same retention time as a cisplatin-urea reaction product and is presumed to be that material. One peak at at ca. 225 has the same retention time as one of the uric acid-cisplatin reaction products. Another peak at 106 s is unidentified. Under these chromatographic conditions, the diaqua hydrolysis product of cisplatin elutes after 1800 s.

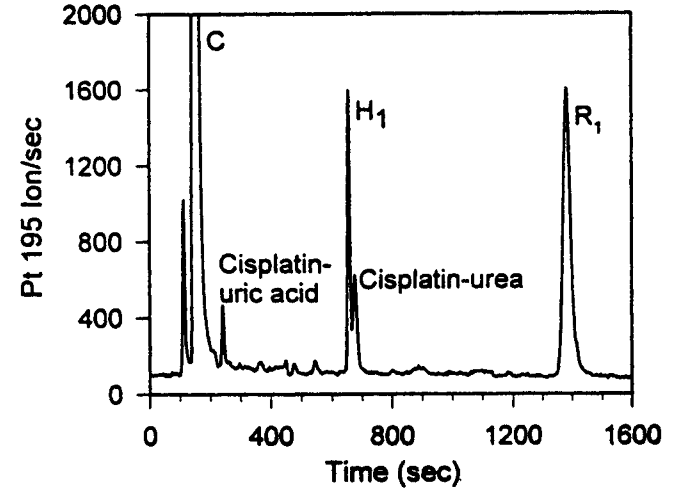

**Figure 7 HPLC separation of urine sample from a lung cancer patient. The patient was given 0.5L saline as hydration therapy (33), followed by a two hour infusion of cisplatin. LC conditions are as in Figure 5. Specific peaks are identified based on retention time.**

We have now examined urine specimens from a number of different patients, using samples taken immediately after drug infusion or three to four weeks after the initial treatment. Figure 8a shows the HPLC separations of samples from four different patients. The dominant platinum containing species in all cases has the retention time of cisplatin. All samples also contain species with the retention times for the monoaqua hydrolysis product and the 1:1 cisplatin-creatinine complex ($R_1$). As with the rat samples, the identity of $R_1$ was confirmed by the addition of the 1:1 reaction mixture to the urine specimen. The peak at approximately 800 s increased in height but did not change in shape.

We have also analyzed the urine of patients approximately three weeks after one dose (immediately prior to the next dose). In these patients, there is still a significant amount of platinum present. Platinum levels immediately after treatment are typically in the 30-50 ppm range, while after three weeks, levels are in the 50-100 ppb range. Figure 8b shows chromatograms of several urine samples taken after three weeks. The upper and middle traces are from one patient, but taken on two different dates. Both chromatograms show several platinum peaks, indicating that significant biotransformation of the cisplatin has occurred during the time following treatment. Many of the platinum species have the same retention times, but the quantitative distribution is not identical. After a three week period all three samples show little, if any, of the native cisplatin, nor of the monoaqua hydrolysis product. The identities of the new platinum species have not been determined.

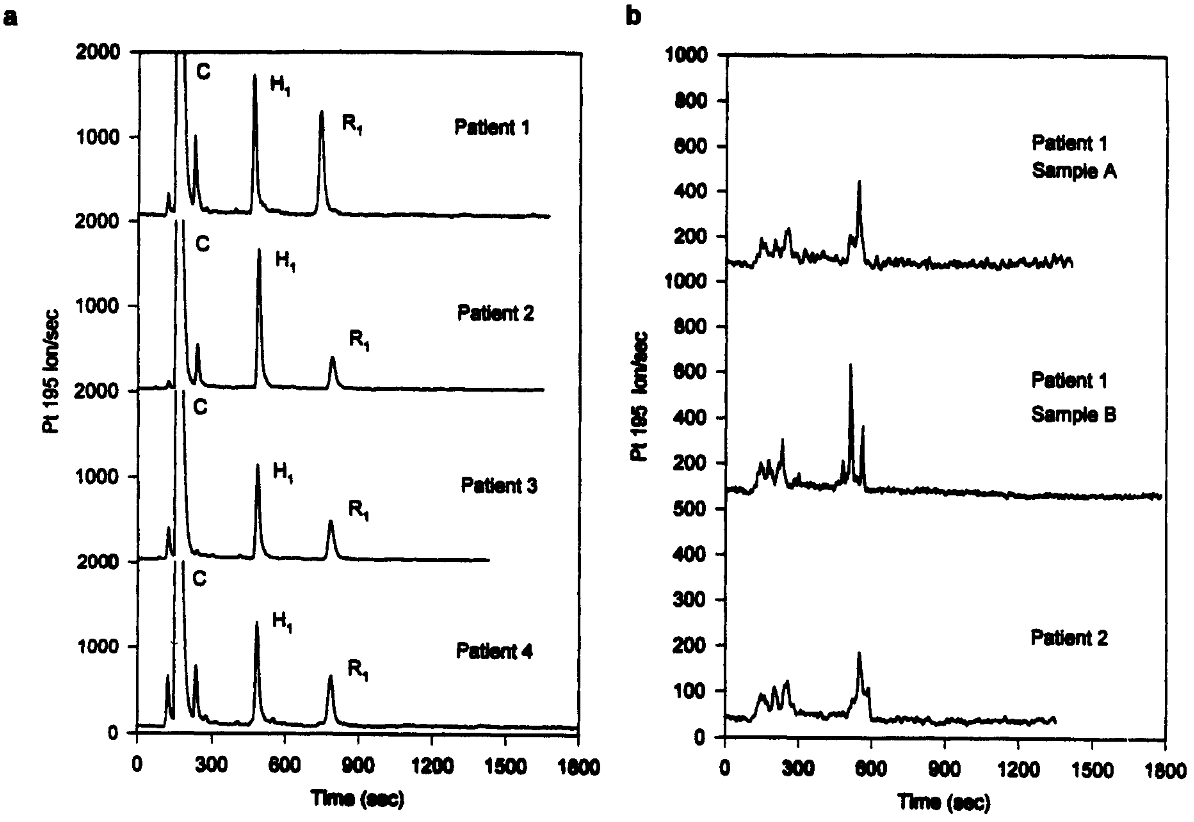

**Figure 8 HPLC separation of urine from cisplatin patients. (a) Samples were taken immediately after cisplatin infusion and (b) samples taken three weeks following treatment. (C) indicates cisplatin, ($H_1$) the monoaqua hydrolysis product, ($R_1$) the 1:1 reaction mixture of cisplatin and creatinine. Mobil phase: 0.1% TFA, 5 mM heptanesulfonate, 10% methanol, pH 2.6-2.7. Column: Ranin Microsorb-MV-$C_{18}$.**

**Carboplatin in Human Urine.** We have begun to use HPLC-ICP-MS to study carboplatin and its biotransformation in patient samples. The HPLC separations of urine from three patients are shown in Figure 9. Specimens analyzed in the left panel were taken immediately following the carboplatin treatment. The right panel shows analyses of urine from the same patients three weeks after treatment. In all three samples immediately after treatment, the main platinum species present is carboplatin. In one of the patients, there is an additional platinum species present, which is not retained by the column. A minor platinum peak is also present in all the samples at approximately 375 s. None of these species correspond to those observed in the urine of the cisplatin patients. In the urine samples collected three weeks after the dose, there is still significant platinum present (150-275 ppb). By this point, significant biotransformation has occurred. Interestingly, in one of the patients, Patient 5, there are platinum species present which have the retention times for cisplatin and the monoaqua hydrolysis product. This result provides evidence that carboplatin can be converted *in vivo* to cisplatin, followed by hydrolysis and presumably other subsequent reactions characteristic of administered cisplatin.

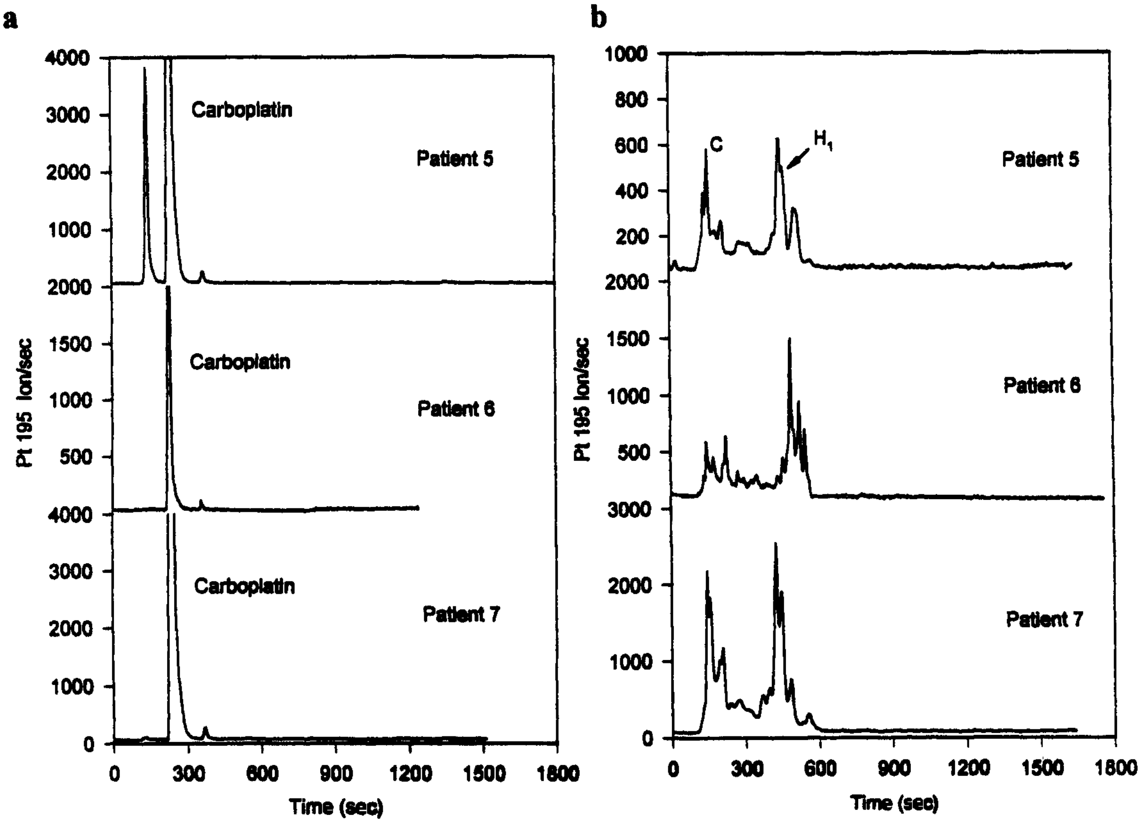

**Figure 9 HPLC separations of urine from patients treated with carboplatin. Samples were taken either immediately following treatment (a) or three weeks after treatment. LC separations are as in Figure 8.**

### Discussion

Clearly the biotransformation chemistry of cisplatin is extremely rich. Others have shown evidence that cisplatin-glutathione products are formed in the body (9). By using HPLC analysis coupled with ICP-MS to provide platinum specific detection, we have shown that a large number of biotransformation products can be formed as a result of *in vitro* reactions with nitrogenous ligands such as creatinine, urea and uric acid. Some of these reaction products are detectable in the urine of patients undergoing cisplatin chemotherapy. Comparison of the chromatograms from samples taken immediately after therapy with those taken weeks later indicates that significant biotransformation continues to occur. In a single individual, the chromatograms taken at different times are qualitatively similar, but there are quantitative differences. Comparison of samples from different individuals reveals some qualitative differences as well.

Comparison of the samples from cisplatin and carboplatin patients illustrates significant differences between the two drugs. The samples from carboplatin patients taken immediately after treatment show fewer biotransformation products, with carboplatin itself being the principal platinum species present. This observation is likely the result of the "relative stability" of the leaving groups of the two compounds (6). Essentially all the Pt detectable by flow injection is recovered on the chromatographic column. The primary metabolite identified in rat urine, a ring-opened adduct of carboplatin-methionine (34) is not present in these patients. The cationic methionine complex should elute after the carboplatin under these chromatographic conditions. In the weeks following treatment, at least one of the carboplatin patients appears to have metabolized the compound into cisplatin, since the chromatogram shows peaks with the retention times for both cisplatin and the monoaqua hydrolysis product (Figure 9b). This is a confirmation of the suggestion that cisplatin is a metabolite of carboplatin (35).

Using HPLC coupled to the ICP-MS for platinum-specific detection will allow us to gain a great deal more insight into the biotransformation chemistry of cisplatin and the next generation agents such as carboplatin. Hopefully these insights will lead to better control of toxicity and higher efficacy for platinum antineoplastic agents.

### ACKNOWLEDGMENTS

This work was supported by a Biomedical Research Support Grant from the NIH. The ICP-MS and FAB-MS were purchased by the Biomedical Chemistry Research Center with funds from an Academic Challenge Grant from the State of Ohio. The authors thank Lisa Steelman for her help in providing patient samples and in maintaining the appropriate records, James Carlson for conducting the FAB-MS studies and Laura Rosenblum and Elwood Brooks for help in measuring the NMR spectra.

### REFERENCES

1. B. Rosenberg, L. VanCamp, J.E. Trosko, V.H. Massour, *Nature* **222**, 385 (1969).
2. B. Rosenberg, *Cancer* **55**, 2303 (1985).
3. G.K. McEvoy, in *AHFS Drug Information* **94**, (American Society of Hospital Pharmacists, 1994), pp 572-580.
4. G. Daugaard and U. Abildgaard, *Cancer Chemother. Pharmacol.* **25**, 1 (1989).
5. C.L. Litterst, *Toxicol. Appl. Pharmacol.* **61**, 99 (1981).
6. H. Calvert, I. Judson, W.J.F. Van der Vijgh, *Cancer Surveys* **17** *Pharmacokinetics and Cancer Chemotherapy,* Imperial Cancer Research Fund, pp 189 (1993).
7. J.C. Ruckdeschel, *Seminars in Oncology,* **21 (suppl. 12)** 114 (1994).
8. P.T Daley-Yates and D.H.C. McBrien, *Cancer Chemother. Pharmacol.* **33**, 3063 (1989).
9. P. Mistry, C. Lee, D.H.C. McBrien, *Cancer Chemother. Pharmacol.* **24**, 73 (1989).
10. C.M. Riley, L.A. Sternson, A.J. Repta, S.A. Slyter, *Anal. Biochem.* **130**, 203 (1983).
11. R.E. Norman, J.D. Ranford, P.J. Sadler, *Inorg. Chem.* **31**, 877 (1992).
12. B. Ottenheeimer and W. Wolf, *Inorg. Chim. Acta* **66**, L41 (1982).
13. S.J. Berners-Price and P.W.J. Kuchel, *Inorg. Biochem.* **38**, 305 (1990).
14. S.R. Burke, *The Composition and Function of Body Fluid*, The C.V.Mosby Company, St. Louis (1980).
15. C.F.G. C.Geraldes, M. Aragon-Salgado, J. Martín-Gil, *Polyhedron* **10**, 799 (1991).
16. F. J. Martín-Gil, J. Martín-Gil -Gil, *Inorg. Chim. Acta* **137**, 131 (1987).
17. P. R. Bontchev, M. Mitewa, G. Gentcheva, *Pure & Appl. Chem.* **61**, 897 (1989).
18. N. Trendafilova, A. P. Kurbakova, I. A. Efimenko, M. Mitewa, P. R. Bontchev, *Spect. Act.* A **47**, 577 (1991).
19. Z. Zhao, W. B.Jones, K.Tepperman, J. G.Dorsey, R. C. Elder, *J. Pharm. Biomed. Anal.* **10**, 279 (1992).
20. Z. Zhao, K. Tepperman, J. G. Dorsey, R.C. Elder, *J Chromatog.* **615**, 83 (1993).
21. L. A. Scott, E. Madan, M. A. Valentovic, *Fundam. Appl. Toxicol.* **12**, 530 (1989).
22. S. G. Matz, K. Tepperman, R. C. Elder, *Anal. At. Spec.* **4**, 767 (1989).
23. E. Segal, J.-B. Le Pecq, *Cancer Res.* **45**, 492 (1985).
24. T.G. Appleton, R.D. Berry, C. A. Davis, J. R. Hall, H. A. Kimlin, *Inorg. Chem.* **23**, 3514 (1984).
25. Rat urine specimens were obtained under a protocol (94-08-30-01) approved by the Institutional Animal Care and Use Committee of the University of Cincinnati.

26. Clinical urine specimens were obtained from patients at Barrett Cancer Center of the University of Cincinnati in accord with human studies protocol #93-3-16-3E.
27. M. Kinoshita, N. Yoshimura, H. Ogata, *J. Chromatog.* **529**, 462 (1990).
28. R. F.Dietrich, M. A. Marletta, G. L. Kenyon, *Org. Magn. Resonance* **13**, 79 (1980).
29. The $^{13}C$ resonances of the 1:2 Pt-creatinine complex obtained in our experiments are similar to those of creatinine provided by Geraldes *et al.* in reference 12, and the resonances of creatinine agree with those of the Pt-creatinine complex in their results.
30. P. T. Beurkens, A. Perales, F. J. Martín-Gil, J. Martín-Gil, *Monatsh. Chem.* **119**, 1189 (1988).
31. W. Cacini, E. A. Harden, K. A. Skau, *Proc. Soc. Exp. Biol. Med.* **203**, 348 (1993).
32. W. Cacini, Y. Singh, *Proc. Soc. Exp. Biol. Med.* **197**, 285 (1991).
33. R.F. Borch, in *Toxicology of the Kidney*, Raven Press, New York, p283 (1993).
34. K.j. Barnham, U. Frey, P. del S. Murdoch, J.D. Ranford, P.J. Sadler, D. Newell *J. Am. Chem. Soc.* **116**, 11175 (1994).
35. N.D. Tinker, H.L. Sharma, C.A McAuliffe, In:*Platinum and Other Metal Coordination Compounds in Cancer Chemotherapy: Proeedings of the Fifth International Symposium*, ed. M. Nicolini, Martinus Nijhoff, Boston, p144 (1987).

**Received: March 12, 1997 - Accepted: April 70, 1997 -**
**Received in revised camera-ready format: April 23, 1997**

